# Ageism in a Latin American Cohort: The Role of Cognition and Gender

**DOI:** 10.1002/alz70858_105342

**Published:** 2025-12-26

**Authors:** Micaela Maria Arruabarrena, Nicolas Corvalan, Florentina Morello Garcia, Greta Keller, Carlos Martinez, Luc¡a Crivelli

**Affiliations:** ^1^ Fleni, Buenos Aires, Buenos Aires, Argentina; ^2^ Institute of Neurosciences (INEU), Fleni‐CONICET, Buenos Aires, Buenos Aires, Argentina; ^3^ Fleni, Buenos Aires, Argentina

## Abstract

**Background:**

Ageism, a form of discrimination based on age‐related stereotypes, encompasses both negative (e.g., older adults as burdensome or incapable) and positive (e.g., older adults as wise or nurturing) stereotypes. According to the Stereotype Embodiment Theory (SET), these cultural stereotypes, internalized throughout life, shape self‐perceptions, behaviors, and health outcomes as individuals age. The objective of this study is to evaluate the differences in perceived ageism between individuals with Mild Cognitive Impairment (MCI) and normal controls (NC). Additionally, it aims to analyze whether men and women perceive ageism differently, considering gender effects and potential interactions with cognitive status.

**Method:**

Our study included 188 participants (57.4% women) aged 60 to 88 years (72.2±6.7). According to the Clinical Dementia Rating (CDR), 136 participants were classified as NC (CDR=0) and 52 as MCI (CDR=0.5). All the participants completed the 8‐item Perceived Ageism Questionnaire (PAQ) to evaluate positive and negative ageism. Generalized linear models assessed the relationship between ageism perceptions and cognitive status, gender, age, and their interactions.

**Result:**

No significant differences in Positive Ageism (*p* = .548) or Negative Ageism (*p* = .079) were found between MCI and NC. In the Positive Ageism model, lower cognitive status (𝛃=‐3.278, SE=0.4657, *p* = .0155) and the interaction between gender and cognitive status (𝛃=2.3821, SE=0.8812, *p* = .0075) were significant predictors. Intragroup comparisons and post‐hoc analysis showed that in the MCI group, men perceived higher Positive Ageism than women (*p* = .028), which was not present in the NC group (*p* = 0.897). In addition, no significant gender differences in Negative Ageism were observed in either the MCI or NC groups.

**Conclusion:**

Our findings highlight distinct patterns in positive and negative ageism according to the interaction between gender and cognitive status. Men with MCI perceive significantly higher Positive Ageism, suggesting that aging in men is perceived as an advantage, reinforcing notions of wisdom and social value. In contrast, women with MCI do not share this positive perception, likely reflecting societal attitudes that devalue aging in women. Future studies should explore these interactions to understand better the impact of ageism on older adults with MCI.

